# *trans*-Cinnamaldehyde Inhibits Microglial Activation and Improves Neuronal Survival against Neuroinflammation in BV2 Microglial Cells with Lipopolysaccharide Stimulation

**DOI:** 10.1155/2017/4730878

**Published:** 2017-10-22

**Authors:** Yan Fu, Pin Yang, Yang Zhao, Liqing Zhang, Zhangang Zhang, Xianwen Dong, Zhongping Wu, Ying Xu, Yongjun Chen

**Affiliations:** ^1^Department of Physiology, School of Basic Medicine, Shanghai University of Traditional Chinese Medicine, 1200 Cailun Road, Shanghai 201203, China; ^2^Department of Clinical and Classic Medicine, School of Basic Medicine, Shanghai University of Traditional Chinese Medicine, 1200 Cailun Road, Shanghai 201203, China; ^3^South China Research Center for Acupuncture and Moxibustion, Medical College of Acu-Moxi and Rehabilitation, Guangzhou University of Chinese Medicine, 232 Waihuan Dong Road, Guangzhou 510006, China

## Abstract

**Background:**

Microglial activation contributes to neuroinflammation and neuronal damage in neurodegenerative disorders including Alzheimer's and Parkinson's diseases. It has been suggested that neurodegenerative disorders may be improved if neuroinflammation can be controlled.* trans*-cinnamaldehyde (TCA) isolated from the stem bark of* Cinnamomum cassia* possesses potent anti-inflammatory capability; we thus tested whether TCA presents neuroprotective effects on improving neuronal survival by inhibiting neuroinflammatory responses in BV2 microglial cells.

**Results:**

To determine the molecular mechanism behind TCA-mediated neuroprotective effects, we assessed the effects of TCA on lipopolysaccharide- (LPS-) induced proinflammatory responses in BV2 microglial cells. While LPS potently induced the production and expression upregulation of proinflammatory mediators, including NO, iNOS, COX-2, IL-1*β*, and TNF-*α*, TCA pretreatment significantly inhibited LPS-induced production of NO and expression of iNOS, COX-2, and IL-1*β* and recovered the morphological changes in BV2 cells. TCA markedly attenuated microglial activation and neuroinflammation by blocking nuclear factor kappa B (NF-*κ*B) signaling pathway. With the aid of microglia and neuron coculture system, we showed that TCA greatly reduced LPS-elicited neuronal death and exerted neuroprotective effects.

**Conclusions:**

Our results suggest that TCA, a natural product, has the potential of being used as a therapeutic agent against neuroinflammation for ameliorating neurodegenerative disorders.

## 1. Introduction

Neuroinflammation is a critical component in both acute and chronic neurodegenerative disorders, exemplified by Alzheimer's disease (AD), Parkinson's disease (PD), ischemia, and traumatic brain injury (TBI) [1–3]. A major characteristic of brain inflammation is microglial activation that accompanies the neurodegenerative process [4]. Microglia, the resident macrophages in the brain, possess properties particularly suitable to mediate cellular inflammatory responses [5]. Microglia are activated in response to brain injury and exposure to lipopolysaccharide (LPS), interferon gamma (IFN-*γ*), or *β*-amyloid [6, 7]. Activated microglia are linked to the release of a number of proinflammatory mediators including nitric oxide (NO), inducible nitric oxide synthase (iNOS), cyclooxygenase-2 (COX-2), interleukin-1 beta (IL-1*β*), tumor necrosis factor alpha (TNF-*α*), chemokines, complements, excitatory amino acids, and reactive oxygen species (ROS) that are thought to contribute to neuronal death, damage, and functional deterioration [8, 9]. Recent studies suggest that nuclear factor kappa B (NF-*κ*B) and mitogen-activated protein kinase (MAPK) signaling pathways including p38, extracellular signal-regulated kinase (ERK), and c-Jun N-terminal kinase (JNK) are involved in the process of inflammation associated with microglial activation. Since microglia-mediated neurotoxicity is a crucial molecular event involved in initiation and progression of neurodegenerative disorders [10], inhibition of microglial activation may thus be a potential therapeutic approach against neuroinflammatory and neurodegenerative disorders [11, 12].

*Cinnamomum cassia (C. cassia)* has been used to treating dyspepsia, gastritis, blood circulation disturbances, and inflammatory disease in China for thousand years. It has been shown to provide a significantly protective effect against glutamate-induced neuronal death [13].* trans*-cinnamaldehyde (TCA), a natural product, is a major bioactive component isolated from the stem bark of* C. cassia *[14–16] and has been reported to possess antitumor, antipyretic, antimicrobial, antidiabetic, and antimutagenic properties [17–20]. Importantly, TCA also has potent anti-inflammatory activity in aging rats, endothelial cells, and monocytes/macrophages [21–23]. Cinnamon and its main constituents including TCA have also been shown to inhibit neuroinflammation in LPS-stimulated BV2 microglial cells [24]. TCA's anti-inflammatory activity is likely to be associated with its suppressive role in toll-like receptor 4- (TLR4-) mediated signaling [25, 26] and is probably mediated by targeting multiple molecular mechanisms because it can inhibit age-related inflammatory NF-*κ*B activation via the NIK/IKK, ERK, and p38 MAPK pathways in aging rats [21]. These results strongly suggested that TCA may represent as an effective anti-inflammatory drug to deter neurodegenerative processes. However, the molecular mechanisms of TCA for being used to protect neuronal damage under neuroinflammation remain unclear.

The objective of this study is to investigate the potential of using TCA to block neuroinflammation and to improve neuronal survival under neuroinflammation. Using LPS-stimulated microglia as a model of activated microglia, our results showed that TCA inhibited the production of NO and IL-1*β* and expression of iNOS and COX-2 by suppressing activation of NF-*κ*B and granted neuroprotective effects evidenced by attenuating microglial neurotoxicity in microglia/neuron coculture system. This study suggests that TCA, a natural product, should be seriously considered as a potential therapeutic agent for a variety of neuroinflammatory and neurodegenerative diseases.

## 2. Materials and Methods

### 2.1. Chemicals and Reagents

Bacterial lipopolysaccharide (LPS) from* E. coli *serotype 0111:B4,* trans*-cinnamaldehyde (TCA), and JSH-23 were purchased from Sigma-Aldrich (St. Louis, MO, USA). LPS was dissolved in sterile saline. TCA and JSH-23 were reconstituted in dimethyl sulfoxide (DMSO). Anti-CD11b monoclonal antibody (OX-42, microglial marker) was from Abcam (Cambridge, UK). Dulbecco's modified Eagle's medium (DMEM), Roswell Park Memorial Institute medium (RPMI), and fetal bovine serum (FBS) were purchased from Gibco (Rockville, MD, USA). All other reagents were purchased from Sigma-Aldrich unless otherwise described.

### 2.2. Cell Culture

The immortalized BV2 murine microglial cell line and rat pheochromocytoma PC12 neuronal cell line were obtained from the Cell Culture Center of the Chinese Academy of Medical Sciences (China). BV2 cells were maintained at DMEM supplemented with 10% heat-inactivated FBS at 37°C in a humidified atmosphere containing 5% CO_2_. PC12 cells were cultured in RPMI supplemented with 10% FBS and 20% horse serum. The growth medium was replenished every third day until confluence. In all experiments, cells were treated with TCA for the indicated times before LPS stimulation.

### 2.3. MTT Cell Viability Assay

The 3-(4,5-dimethylthiazol-2-yl)-2,5-diphenyltetrazolium bromide (MTT) reduction assay was used to determine cell viability. Briefly, BV2 cells (2 × 10^4^ cells/well) were plated in 96-well plates and then subjected to various treatment. To determine cell viability, 0.5 mg/ml MTT solution was added to cells for 4 h. Formed MTT formazan was solubilized with DMSO and quantified at 540 nm using a microplate reader (Synergy 2, BioTek Instruments, Inc., Winooski, VT, USA) and results are expressed as the percentage of cells in treated groups over the untreated Control. Each assay was carried out in triplicate.

### 2.4. Nitrite Quantification

BV2 cells were plated at 2 × 10^4^ cells/well in 96-well plates. Cells were stimulated with LPS (0.1 *μ*g/ml) for 24 h after pretreatment of varying concentrations of TCA. Amount of nitrite in cell culture media was analyzed by Griess reaction as previously described [27] as used to assess NO production. Briefly, 50 *μ*l of cell supernatant was mixed with an equal volume of Griess reagent (1% sulfanilamide in water and 0.1% N-1-naphthylethylenediamine dihydrochloride in 5% phosphoric acid) and incubated at room temperature for 10 min followed by measuring the absorbance at 550 nm with the aid of a microplate reader. The data are the representative of three or more independent experiments.

### 2.5. Western Blot Analysis

To prepare lysates, BV2 cells were cultured in the presence or absence of LPS (0.1 *μ*g/ml) or LPS + TCA (10 *μ*M) for varying times and then lysed in ice cold RIPA buffer [150 mM NaCl, 50 mM Tris-HCl pH 7.4, 1 mM EDTA, 1% Triton X-100, 0.1% SDS, 1% sodium deoxycholate, 2 mM sodium orthovanadate (Na_3_VO_4_), and 1 mM sodium fluoride (NaF)], supplemented 1 mM phenylmethanesulfonyl fluoride (PMSF), and inhibitors of protease and phosphatase (10 *μ*g/ml each of aprotinin, leupeptin, and pepstatin A). The nuclear proteins were then extracted using Nuclear Extraction Reagents kit (Keygen BioTECH, Nanjing, Jiangsu, China). An aliquot of 20 *μ*g of cytosol and nuclear protein was electrophoresed on 10% and 12% sodium dodecyl sulfate-poly-acrylamide gel electrophoresis (SDS/PAGE) gels and then transferred to a nitrocellulose membrane (Amersham Biosciences, Buckinghamshire, UK) followed by incubation with primary antibodies. Primary antibodies used for this studies are anti-iNOS (Cell Signaling Technology, Danvers, MA, USA), anti-COX-2 (CST), anti-NF-*κ*Bp65 (CST), anti-p-I*κ*B*α* (Santa Cruz Biotechnology, Dallas, Texas, USA), anti-I*κ*B*α* (Santa Cruz), anti-*β*-actin (CST), and anti-Histone 3 (ProteinTech, Chicago, IL, USA). The intensities of protein bands were quantified by Image Quant software (Tanon, Shanghai, China). The relative protein level was normalized to *β*-actin or Histone 3.

### 2.6. Quantitative RT-PCR (qRT-PCR)

BV2 cells (2 × 10^6^ cells/well in 6-well plate) used for qRT-PCR analysis were stimulated with LPS (0.1 *μ*g/ml) in the presence or absence of TCA (10 *μ*M) for varying times. Total RNA was isolated with TRIzol reagent (Invitrogen, Carlsbad, CA, USA) according to the manufacturer's instructions and reverse transcribed using PrimeScript™ RT reagent kit with gDNA Eraser (Takara Bio Inc. Otsu, Shiga, Japan) followed by qRT-PCR. The primer sequences were included in the following:

  iNOS (Forward, 5′-CACCTTGGAGTTCACCCAGT-3′; Reverse, 5′-ACCACTCGTACTTGGGATGC-3′)
  IL-1*β* (Forward, 5′-CAGGCAGGCAGTATCACTCA-3′; Reverse, 5′-AGCTCATATGGGTCCGACAG-3′)
  TNF-*α* (Forward, 5′-GAACTGGCAGAAGAGGCACT-3′; Reverse, 5′-AGGGTCTGGGCCATAGAACT-3′)
  COX-2 (Forward 5′-GTCTGGTGCCTGGTCTGATGA-3′; Reverse 5′-TGGTAACCGCTCAGGTGTTG-3′)
 
*β*-Actin (Forward, 5′-AGCCATGTACGTAGCCATCC-3′; Reverse, 5′-TCTCAGCTGTGGTGGTGAAG-3′).

### 2.7. Enzyme-Linked Immunosorbent Assay (ELISA)

BV2 cells (2 × 10^4^ cells/well in 96 well plates) were treated with LPS in the presence or absence of TCA (10 *μ*M) for 6 h. To determine the amount of IL-1*β* and TNF-a secreted, the supernatants of the cells were collected and analyzed using commercially available enzyme-linked immunosorbent assay kits purchased from Invitrogen according to manufacturer's protocol. Experiments were repeated at least three times.

### 2.8. Transfection and Dual Luciferase-Reporter Assay

For measurement of NF-*κ*B transcriptional activity, BV-2 cells were seeded at a density of 5 × 10^4^ cells/well in a 24-well plate. The triplicate wells were cotransfected with a mixture of plasmid (pNF-*κ*B-luc reporter plasmid and PRL-TK internal Control plasmid) using lipofectamine transfection reagent according to manufacturer's specifications (Promega, Madison, WI, US). After 24 h transfection, the cells were pretreated with TCA (10 *μ*M) for 2 h followed by LPS (0.1 *μ*g/ml) stimulation for 6 h. The cells were harvested in lysis buffer and were analyzed for luciferase activity using the Dual Luciferase Reporter Assay System (Promega). The firefly luminescence was quantified, standardized to* Renilla* expression and reported as relative activity.

### 2.9. Immunofluorescence Staining

BV2 cells were treated with LPS in the presence or absence of TCA (10 *μ*M) for 24 h in 24-well plate followed by three times with PBS. The medium was removed and cells were fixed with ice cold 4% paraformaldehyde in 0.1 M phosphate buffer (PB) for 15 min. Cells were permeabilized with 0.2% Triton X-100 for 10 min followed by blocking with 10% normal donkey serum (Jackson ImmunoResearch Lab, West Grove, PA, USA) for 30 min at room temperature. Fixed cells were incubated with anti-CD11b monoclonal antibody (1 : 500) in PBS containing 0.1% Triton X-100 overnight at 4°C and then Alexa Fluor 594-conjugated donkey anti-mouse secondary antibody (1 : 500; Invitrogen) for 1 h at room temperature. The nuclei were counterstained using DAPI. Images of microglia were visualized under an Axiovert 40 CFT visible/fluorescence microscope (Carl Zeiss, Oberkochen, Germany).

### 2.10. Cytotoxicity Assay in Microglia/Neuron Coculture

BV2 cells were pretreated with or without TCA (10 *μ*M) for 2 h followed by LPS (0.1 *μ*g/ml) stimulation. LPS-stimulated BV2 cells were added to transwell inserts (pore size 0.4 *μ*m) while PC12 cells were plated in the underwells. In such coculture system, cells in the transwell inserts can communicate with cells in the underwells through the semipermeable membrane without direct cell contact. After 24 h coculture, MTT assay was performed to evaluate neuronal cell viability. The experimental timeline is shown in Figure 6(a).

### 2.11. Statistical Analysis

Data were presented as the mean ± SEM derived from three or more independent experiments. Statistical analyses were performed using one-way or two-way ANOVA, followed by Holm-Bonferroni post hoc test. The differences were considered statistically significant when *P* < 0.05.

## 3. Results

### 3.1. Effects of TCA on NO Production and Cytotoxicity in LPS-Stimulated BV2 Cells

**Figure 1:**
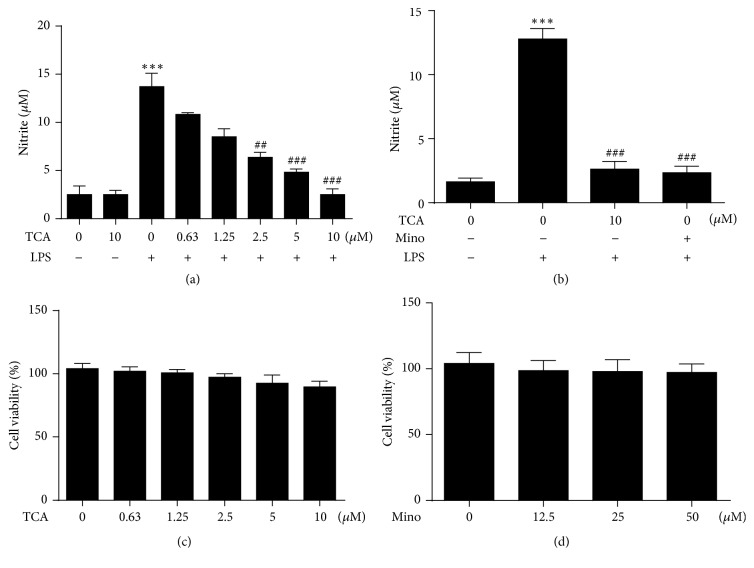
*Effects of TCA on NO production and cell viability in LPS-stimulated BV2 cells.* (a) BV2 cells were pretreated with varying concentration of TCA for 2 h and then stimulated with 0.1 *μ*g/ml LPS for 24 h. Griess assays were performed to measure the amount of nitrite in media of BV2 cells. (b) The inhibitory effects of TCA on NO production were compared to minocycline (Mino) in LPS-stimulated BV2 cells. Cell viability was determined by MTT assay following treatment with TCA (c) and Mino (d). The results are expressed as the percentage of surviving cells over the untreated Control. Each value indicates the mean ± SEM from three independent experiments. ^*∗*^*P* < 0.05, ^*∗∗*^*P* < 0.01, and ^*∗∗∗*^*P* < 0.001 versus untreated Controls; ^#^*P* < 0.05, ^##^*P* < 0.01, and ^###^*P* < 0.001 versus LPS alone.

Since NO production is an excellent indicator of inflammatory response, we evaluate the effects of TCA on LPS-induced NO production in BV2 cell lines. Griess assay showed that TCA dose-dependently diminished LPS-induced NO production in BV2 cells (Figure 1(a)). In a parallel experiment, we compared the effectiveness of TCA to block LPS-induced NO production with the well-known microglial deactivator minocycline (Mino) [28, 29]. TCA at dose of 10 *μ*M displayed similar level of inhibition seen with 50 *μ*M minocycline in BV2 lines (Figure 1(b)). Moreover, to determine the cytotoxic effects of TCA and minocycline on BV2 cells, cells were treated with varying concentrations of TCA and minocycline for 24 h. MTT assay showed that TCA up to 10 *μ*M and minocycline 50 *μ*M displayed no significant effects on cell viability in BV2 cells (Figures 1(c) and 1(d)). Since TCA is not toxic to BV2 lines up to 10 *μ*M, these results suggest that TCA at the concentration of 2.5–10 *μ*M can be safely used to block LPS-induced inflammatory responses in BV2 cells.

### 3.2. Effects of TCA on the Protein Levels of Proinflammatory Mediators in LPS-Stimulated BV2 Cells

**Figure 2:**
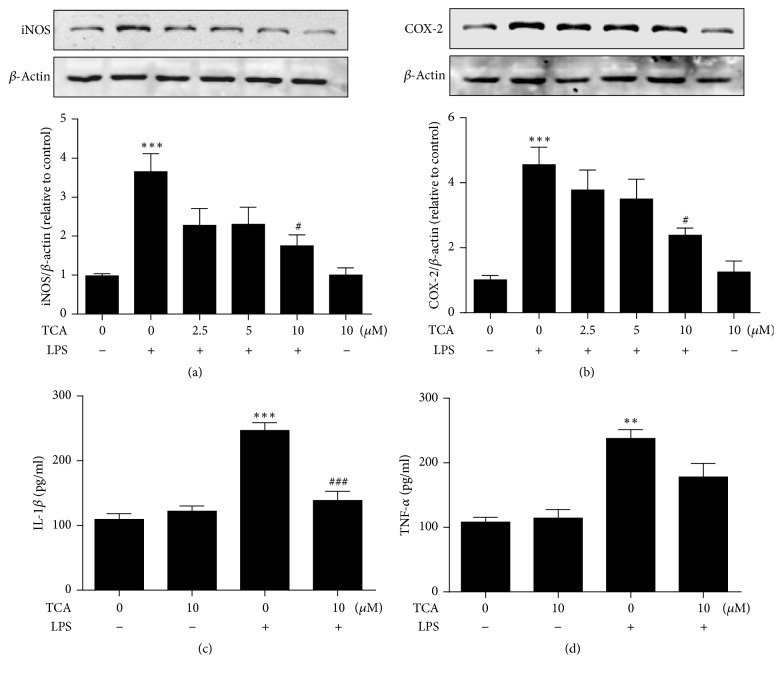
*Effects of TCA on the protein levels of iNOS, COX-2, IL-1β, and TNF-α in LPS-stimulated BV2 cells.* BV2 cells were pretreated with 2.5–10 *μ*M TCA for 2 h prior to 0.1 *μ*g/ml LPS stimulation. After 24 h, cells were lysed and cell lysates were subjected to Western blot to detect iNOS (a) and COX-2 (b) expression. The protein levels of iNOS and COX-2 were standardized based on the respective level of *β*-actin protein. Value was expressed as relative changes in comparison to Control, which was set to 1. Supernatants were collected at 6 h after LPS stimulation and subjected to ELISA to measure the amount of IL-1*β* (c) and TNF-*α* (d) secreted by BV2 cells with TCA 10 *μ*M pretreatment. Each value indicates the mean ± SEM from three independent experiments. ^*∗*^*P* < 0.05, ^*∗∗*^*P* < 0.01, and ^*∗∗∗*^*P* < 0.001 versus untreated Controls; ^#^*P* < 0.05, ^##^*P* < 0.01, and ^###^*P* < 0.001 versus LPS alone.

To clarify molecular mechanism associated with the suppressive effects of TCA on LPS-induced production of proinflammatory mediators, we analyzed the protein levels of iNOS, COX-2, IL-1*β*, and TNF-*α* in BV2 cells stimulated by LPS. Western blot analysis showed that significant upregulation of iNOS and COX-2 expression could be detected in BV2 cells upon LPS stimulation for 24 h, while pretreatment of TCA at dose of 2.5 *μ*M and 5 *μ*M did not significantly prevent LPS-induced upregulation of iNOS and COX-2 expression. Instead, TCA at dose of 10 *μ*M led to a dramatic reduction of iNOS and COX-2 expression at 24 h after LPS stimulation compared to LPS-stimulated microglia without TCA pretreatment (Figures 2(a) and 2(b)). Similar effect was observed with the levels of IL-1*β* release judged by ELISA analysis. ELISA showed that LPS stimulation for 6 h more than doubled the levels of TNF-*α* and IL-1*β* release in BV2 cells, while pretreatment of TCA at 10 *μ*M almost completely abolished LPS-induced IL-1*β* release (Figure 2(c)). TCA pretreatment did not affect the secretion of TNF-*α* in LPS-stimulated BV2 cells (Figure 2(d)).

### 3.3. Effects of TCA on the mRNA Expression of Proinflammatory Mediators in LPS-Stimulated BV2 Cells

**Figure 3:**
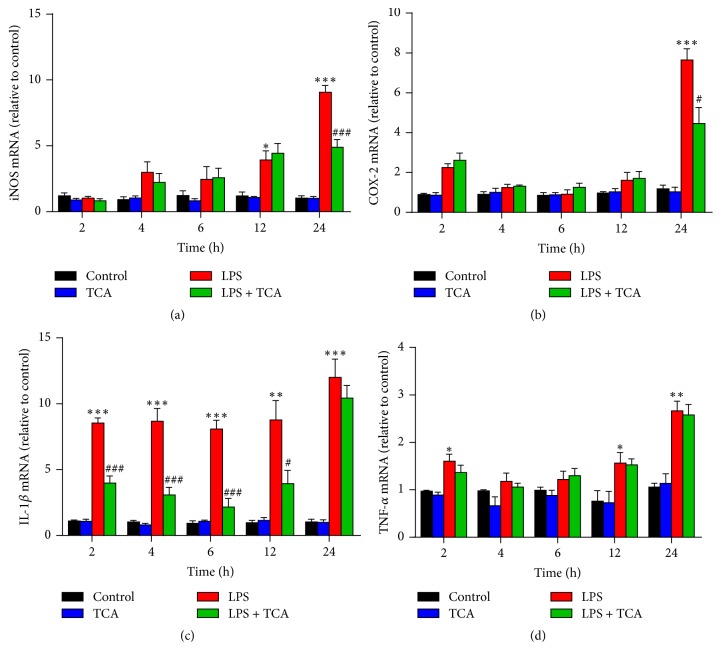
*Effects of TCA on iNOS, COX-2, IL-1β, and TNF-α mRNA in LPS-stimulated BV2 cells.* BV2 cells were stimulated with LPS (0.1 *μ*g/ml) with or without TCA (10 *μ*M) pretreatment for varying times. Total RNA was isolated and subjected to quantitate the levels of iNOS (a), COX-2 (b), IL-1*β* (c), and TNF-*α* (d) mRNA by qRT-PCR analysis. Values are expressed as fold change over the respective Controls. Each value indicates the mean ± SEM from three independent experiments. ^*∗*^*P* < 0.05, ^*∗∗*^*P* < 0.01, and ^*∗∗∗*^*P* < 0.001 versus untreated Controls; ^#^*P* < 0.05, ^##^*P* < 0.01, and ^###^*P* < 0.001 versus LPS alone.

Because of the importance of proinflammatory mediators in chronic inflammation, we examined the effects of TCA on LPS-induced mRNA expression of proinflammatory factors, including iNOS, COX-2, IL-1*β*, and TNF-*α*, in BV2 cells by qRT-PCR analysis. The results showed that LPS stimulation significantly increased the mRNA expression of iNOS, COX-2, IL-1*β*, and TNF-*α* at varying times (Figure 3), while pretreatment of TCA blocked LPS-induced iNOS and COX-2 mRNA at a later time point (after 24 h, Figures 3(a) and 3(b)) and suppressed the upregulation of IL-1*β* mRNA at early time point (before 6 h, Figure 3(c)) but displayed no effects on TNF-*α* mRNA in LPS-stimulated BV2 cells (Figure 3(d)).

### 3.4. TCA Recovers LPS-Induced Morphological Alteration in BV2 Cells

**Figure 4:**
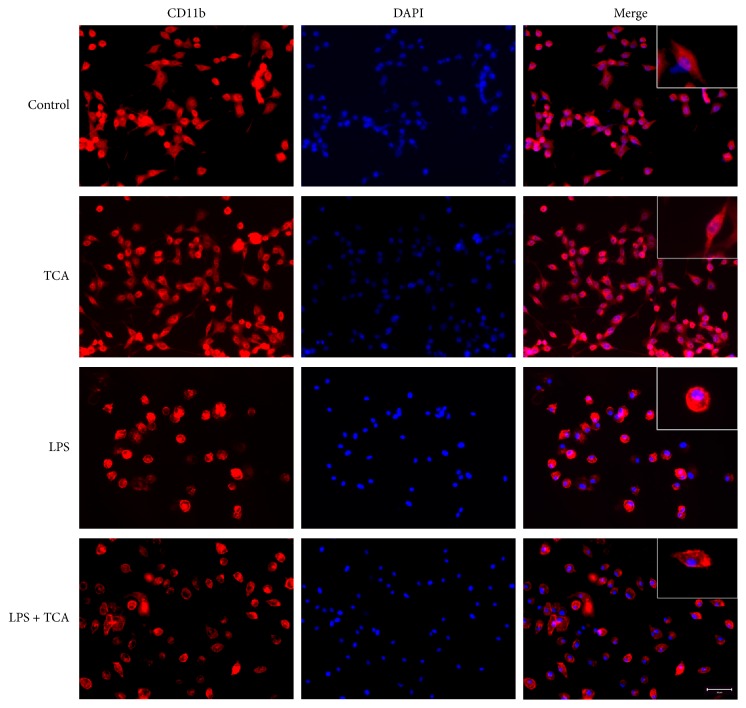
*Effects of TCA on LPS-induced morphological alteration in BV2 cells*. Immunostaining of BV2 cells was performed by using anti-CD11b monoclonal antibody (red) in the presence of DAPI (blue). Staining was visualized under a fluorescence microscopy. Magnification is 20x and scale bar is 100 *μ*m. Insets show high-magnification representative BV2 cells. Control: untreated BV2 cells. Treatment condition: TCA: TCA (10 *μ*M) treatment alone. LPS: LPS (0.1 *μ*g/ml) stimulation alone. LPS + TCA: combined treatment of LPS (0.1 *μ*g/ml) and TCA (10 *μ*M).

Since the morphological alteration of microglia can be observed upon inflammatory condition, we treated BV2 cells with LPS (0.1 *μ*g/ml) in the absence or presence of TCA (10 *μ*M) for 24 h. Anti-CD11b immunofluorescence staining analysis showed that resting BV2 cells exhibited elongated cell bodies and highly ramified processes. In contrast, BV2 cells exposed to LPS displayed larger spherical cell bodies and retracted processes, indicating a morphological transformation into amoeboid/activated. However, TCA pretreatment partially blocked LPS-induced morphological alteration and microglial activation, and short ramified processes were observed in BV2 cells (Figure 4).

### 3.5. Inhibitory Effects of TCA on NF-*κ*B Activation and I*κ*B*α* Phosphorylation in LPS-Stimulated BV2 Cells

**Figure 5:**
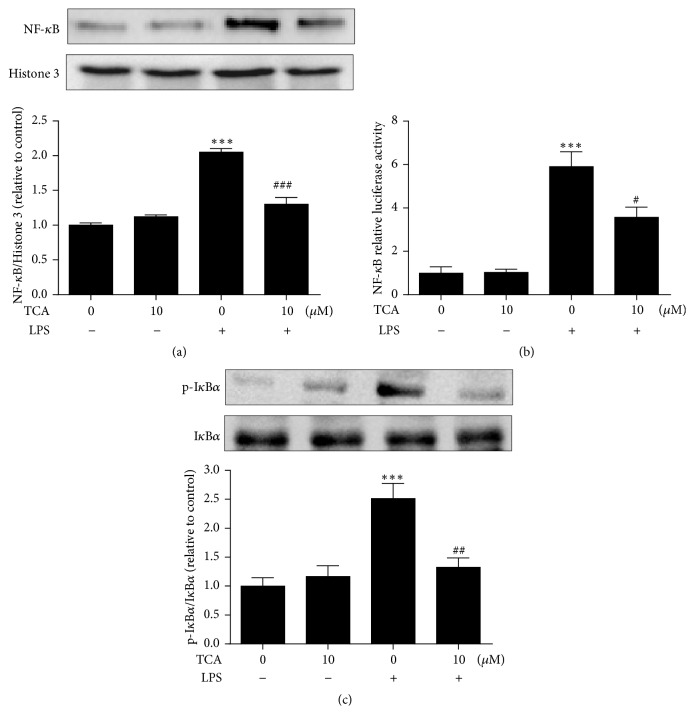
*Inhibitory effects of TCA on NF-κB activation and IκBα phosphorylation in LPS-stimulated BV2 cells.* (a) Total nuclear protein was extracted to detect NF-*κ*B expression followed by Western blot using an anti-NF-*κ*Bp65 antibody. Quantification of protein band densities was normalized to the corresponding levels of Histone 3. (b) Effects of TCA on LPS-induced NF-*κ*B luciferase activity in BV2 cells. (c) Total cytosolic protein was extracted to detect p-I*κ*B*α* expression, which were normalized with the levels of total I*κ*B*α*. Value was expressed as relative changes in comparison to Controls, which was set to 1. Data are the mean ± SEM from three independent experiments. ^*∗*^*P* < 0.05, ^*∗∗*^*P* < 0.01, and ^*∗∗∗*^*P* < 0.001 versus untreated Controls; ^#^*P* < 0.05, ^##^*P* < 0.01, and ^###^*P* < 0.001 versus LPS alone.

The activation of NF-*κ*B by LPS stimulation can induce the expression of proinflammatory mediators, which contribute to the pathogenesis of the inflammatory process [27]. We investigated the regulation of NF-*κ*B activation by TCA using Western blot of nuclear NF-*κ*B analysis and Dual Luciferase-reporter assay. The transfected BV2 cells were pretreated with TCA (10 *μ*M) for 2 h followed by the LPS (0.1 *μ*g/ml) stimulation for 6 h. We examined the effects of TCA on the nuclear translocation of the p65 subunit of NF-*κ*B in LPS-stimulated BV2 cells. The NF-*κ*B activation was induced by LPS stimulation. It also caused the nuclear translocation of the p65 (cytosol), subunit of NF-*κ*B. The above process was dramatically suppressed by TCA (Figure 5(a)). Further, the cells were cotransfected with pNF-*κ*B-luc reporter and PRL-TK plasmid. After transfection, cells were treated with TCA (10 *μ*M) for 2 h before the 6 h LPS stimulation (0.1 *μ*g/ml), and then NF-*κ*B transcriptional activity was determined and expressed as relative luciferase activity (RLU). As shown in Figure 5(b), NF-*κ*B transcriptional activity was significantly enhanced by LPS stimulation, while the enhancement of NF-*κ*B activity was inhibited by TCA pretreatment. In addition, we also investigated the effects of TCA on the cytosolic expression of p-I*κ*B*α* and I*κ*B*α* in LPS-stimulated BV2 cells.  Figure 5(c) showed that pretreatment of TCA decreased LPS-induced phosphorylation of I*κ*B*α*, which further indicated that the subsequent NF-*κ*B inactivation was induced by TCA in LPS-stimulated BV2 cells. Together, these results suggested that the inhibition of NF-*κ*B activation by TCA may be the mechanism responsible for the suppression of proinflammatory mediators in LPS-stimulated BV2 cells.

### 3.6. Attenuation of Microglial Neurotoxicity in the Microglia/Neuron Coculture by TCA Pretreatment

**Figure 6:**
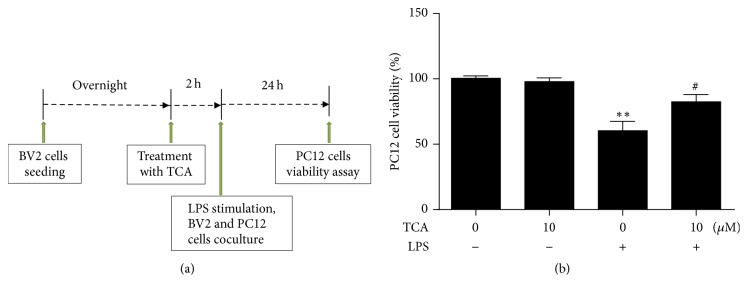
*TCA attenuated microglial neurotoxicity in the microglia/neuron coculture.* BV2 cells were cocultured with PC12 cell using transwell system. (a) The experimental timeline for microglia/neuron coculture. BV2 cells were pretreated with or without TCA (10 *μ*M) for 2 h and then stimulated with LPS (0.1 *μ*g/ml) prior to coculture. (b) After 24 h of coculture period, transwell inserts were removed and MTT assay was performed to determine the viability of PC12 cells in the underwells. Data are the mean ± SEM from three independent experiments. ^*∗*^*P* < 0.05, ^*∗∗*^*P* < 0.01, and ^*∗∗∗*^*P* < 0.001 versus untreated Controls; ^#^*P* < 0.05, ^##^*P* < 0.01, and ^###^*P* < 0.001 versus LPS alone.

Excessively activated microglia through the release of various proinflammatory mediators are well recognized as a major contributing factor to neuronal degeneration [30]. We thus carried out microglia/neuron coculture system to investigate the potential neuroprotective effects of TCA on microglial neurotoxicity (Figure 6(a)). We plated BV2 cells in the inserts and PC12 cells in the underwells, which have been shown to allow the free exchange of proinflammatory cytokines without direct contact between BV2 and PC12 cells [31, 32]. The coculture experiments revealed that LPS-stimulated BV2 cells decreased the viability of PC12 cells while unstimulated BV2 cells did not. However, pretreatment of PC12 cells with TCA significantly improved cell viability (Figure 6(b)). These results implicate that TCA may possess the capability to protect neuronal cells from being damaged by suppressing activated microglia-mediated inflammation.

### 3.7. The Effects of TCA and JSH-23 on NO Production and Neurotoxicity in LPS-Stimulated BV2 Cells and Microglia/Neuron Coculture

**Figure 7:**
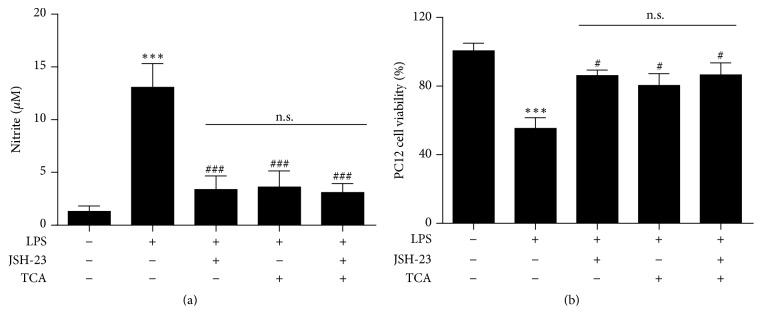
TCA inhibited LPS-induced NO production in BV2 cells and neurotoxicity in microglia/neuron coculture by intercepting the NF-*κ*B signaling pathway. (a) BV2 cells were stimulated with LPS (0.1 *μ*g/ml) in the absence or presence of TCA (10 *μ*M), JSH-23 (25 *μ*M), or TCA + JSH-23 for 24 h followed by Griess assay to measure the level of nitrite in medium. (b) MTT assay was performed to analyze PC12 cells viability in microglia/neuron coculture with LPS stimulation in the absence or presence of TCA (10 *μ*M), JSH-23 (25 *μ*M), or TCA + JSH-23 for 24 h. Data are the mean ± SEM from three independent experiments. ^*∗*^*P* < 0.05, ^*∗∗*^*P* < 0.01, and ^*∗∗∗*^*P* < 0.001 versus untreated Controls; ^#^*P* < 0.05, ^##^*P* < 0.01, and ^###^*P* < 0.001 versus LPS alone.

As NF-*κ*B signaling pathway may be involved in the inhibitory effects of TCA on proinflammatory mediators in LPS-stimulated BV2 cells, we subsequently investigated the effects of JSH-23, an inhibitor of NF-*κ*B nuclear translocation, on NO production in LPS-stimulated BV2 cells and neurotoxicity in microglia/neuron coculture. Griess assay showed that JSH-23 decreased the level of NO production in LPS-stimulated BV2 cells. Moreover, MTT assay showed that JSH-23 could significantly upregulate neuronal viability against microglial neurotoxicity in microglia/neuron coculture. Since the addition of TCA did not further reduce NO production (Figure 7(a)) and improve neuronal survival with JSH-23 treatment (Figure 7(b)), these results indicate that TCA prevents neuroinflammatory-induced neuronal damage by intercepting the NF-*κ*B signaling pathway.

## 4. Discussion

Under the neurodegenerative condition, microglia can be activated and release a variety of neurotoxic and proinflammatory mediators such as iNOS, COX-2, IL-1*β*, and TNF-*α* which are associated with severe neuronal damage and progression of neuroinflammation [9, 33]. Neuroinflammation is a host defense mechanism for protecting the central nervous system (CNS) against aging, infection, and injury. However, sustained neuroinflammatory responses can contribute to neuronal damage and memory impairment in neurodegenerative disorders. Therefore, the suppression of microglial activation and subsequent neuroinflammation have been considered as an effective therapeutic strategy to alleviate the progression of neuroinflammation-mediated neurodegenerative disorders.

Both* in vitro* and* in vivo *studies have demonstrated anti-inflammatory potential of TCA, the major constituent from* C. cassia *[21, 34]. In this study, we investigated the possibility of using TCA to prevent neuroinflammation-caused neuronal damage. Our results showed that TCA was capable of decreasing LPS-induced NO production dose-dependently without eliciting cytotoxicity in BV2 cells (Figures 1(a) and 1(c)). Moreover, pretreatment of TCA (10 *μ*M) significantly prevented LPS-induced upregulation of iNOS and COX-2 expression in BV2 cells after 24 h. TCA has been previously reported to reduce the levels of iNOS and COX-2 in the LPS-stimulated BV2 cells [24, 35]. However, the dose of TCA used in our studies is different from other previous reports. We found that TCA at dose of 10 *μ*M, but not 2.5 *μ*M or 5 *μ*M, dramatically reduced the levels of iNOS and COX-2 expression in the 24 h after LPS-stimulated BV2 cells (Figures 2(a) and 2(b)). Moreover, we also found that TCA could display cytotoxicity above dose of 20 *μ*M (results not shown).

Furthermore, we determined the effects of TCA (10 *μ*M) on the mRNA expression of iNOS and COX-2 in LPS-stimulated BV2 cells for varying times (Figure 3). We found that pretreatment of TCA blocked LPS-induced iNOS and COX-2 mRNA at later time point (after 24 h, Figures 3(a) and 3(b)). The same inhibitory effects of TCA were observed on IL-1*β* mRNA and production in LPS-stimulated BV2 cells (Figures 2(c) and 3(c)), but TCA displayed no effects on TNF-*α* mRNA and production in LPS-stimulated microglia (Figures 2(d) and 3(d)). Subsequently, we observed that TCA also prevented the conversion of resting microglia into activated ones in LPS-stimulated BV2 cells (Figure 4). The suppressive effects of TCA on microglial activation by recovering morphologic change are most likely linked to its ability to block LPS-induced proinflammatory mediators expression. These results are consistent with our previous reports that TCA treatment can suppress primary microglial activation and improve memory deficits in neurodegenerative disease models [36].

However, the molecular mechanisms associated with TCA's suppressive effects on neuroinflammation were poorly elucidated. In present study, we further investigated the regulation of NF-*κ*B signaling pathway by TCA pretreatment using a reporter gene assay and Western blot analysis. NF-*κ*B is an important transcription factor, which is activated by inflammatory stimulation that are associated with the regulation of cell survival and expression of proinflammatory mediators and enzymes such as iNOS, COX-2, IL-1*β*, and TNF-*α* [37, 38]. NF-*κ*B and inhibitory I*κ*B protein are complexed in the cytoplasm. Inflammatory stimulation causes phosphorylation and subsequent degradation of I*κ*B. Free NF-*κ*B (p65 subunit) enters the nucleus and mediates many proinflammatory mediators. Intranuclear blockage of NF-*κ*B has been demonstrated to suppress the expression of proinflammatory mediators. Our results also indicated LPS-induced upregulation of I*κ*B phosphorylation, NF-*κ*B p65 nuclear translocation, and NF-*κ*B luciferase activity. Again, this process was inhibited by TCA pretreatment (Figure 5). It is consistent with a previous study where NF-*κ*B signaling was downregulated by TCA in various cell culture models including RAW264.7 and TLR4-expressing HEK293 [22]. These results suggested that TCA decreased the production of downstream proinflammatory mediators in BV-2 cells, the mechanism of which at least in part might involve the inhibition of NF-*κ*B activation.

Microglial activation has been suggested as a major cause of neuronal damage [27]. The most likely mechanism behind microglial activation-induced neuroinflammation and neuronal death is the excessive production of proinflammatory mediators that are neurotoxic [39–41]. For example, iNOS expression and NO production are upregulated in activated microglia [42, 43]. Anti-inflammatory agents have been shown to inhibit microglial activation and production of proinflammatory mediators. Importantly, these agents are able to attenuate neuronal degeneration and thus exert neuroprotective effects [44, 45]. The fact that TCA is able to reduce proinflammatory mediators expression in LPS-stimulated BV2 cells suggests that TCA may grant protective effects to neuronal cells under inflammation. In this study, our data showed that LPS-stimulated BV2 cells conferred significant toxicity to PC12 neuronal cells in a transwell-based coculture system. However, pretreatment of TCA significantly improved PC12 cell survival (Figure 6). To determine whether the NF-*κ*B signaling pathway is involved in the neuroprotective effects of TCA in LPS-stimulated microglia/neuron coculture, we treated LPS-stimulated microglia with specific inhibitor, JSH-23, to block NF-*κ*B signaling pathway followed by improving PC12 cells survival. Since combined treatment of JSH-23 and TCA had similar effects on NO production and neuronal survival as JSH-23 used alone, we conclude that TCA inhibits microglial neurotoxicity by interfering with NF-*κ*B signaling pathway (Figure 7(b)). This observation raises the possibility of using TCA as a natural product to deter neurodegenerative process.

## 5. Conclusions

**Figure 8:**
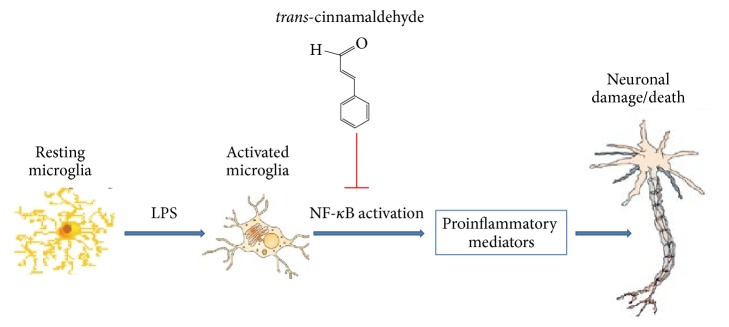
Schematic diagram representing TCA's inhibition of microglial activation and improvement of neuronal survival by blockage of NF-*κ*B activation.

As illustrated in the summary diagram (Figure 8), we provide the evidences that natural product TCA can protect neuronal damage under neuroinflammatory condition and TCA accomplishes its role by suppressing microglial activation and proinflammatory mediators expression via blocking NF-*κ*B signaling pathway in LPS-stimulated BV2 cells. Our study suggests that TCA, a natural product, may be represented as a potential therapeutic agent for ameliorating neuroinflammation-mediated neurodegenerative diseases.

## Additional Points

*Highlights.* TCA suppresses microglial activation and proinflammatory mediators expression via blocking NF-*κ*B signaling pathway in LPS-stimulated BV2 cells. TCA can protect neuronal damage under neuroinflammatory condition. TCA is a potential therapeutic natural product for ameliorating neuroinflammation-mediated neurodegenerative diseases.
